# SuperPath® vs*.* direct anterior approach: A retrospective comparison between two minimally invasive approaches in total hip arthroplasty

**DOI:** 10.1007/s00132-022-04310-0

**Published:** 2022-10-07

**Authors:** André Busch, Alexander Wegner, Dennis Wassenaar, Daniel Brandenburger, Marcel Haversath, Marcus Jäger

**Affiliations:** 1 Department of Orthopedics, Trauma and Reconstructive Surgery, Philippusstift Essen, Essen Hülsmannstraße 17, 45355 Essen, Germany; 2 Chair of Orthopedics and Trauma Surgery, University of Duisburg – Essen, Essen, Germany; 3 Departments of Orthopedics, Trauma and Reconstructive Surgery, St. Marien Hospital Mülheim, Kaiserstraße 50, 45468 Mülheim a. d. Ruhr, Germany

**Keywords:** Total hip arthroplasty, Minimal-invasive approach, Implant position, Offset, Center of rotation, Hüfttotalendoprothese, Minimal-invasiver Zugang, Implantatposition, Offset, Drehzentrum

## Abstract

**Objective:**

Minimally invasive approaches are subject to controversy in orthopedic surgery. The aim of the current study was to compare the radiographic parameters between two minimally invasive approaches in total hip arthroplasty.

**Material and methods:**

Between January 2018 and February 2019, the radiographic parameters of 80 patients undergoing total hip arthroplasty via minimally invasive approaches (DAA: *n* = 40; SuperPath® SP: *n* = 40) have been measured. The radiographic analysis was performed with digital software tool mediCad® (HECTEC™ GmbH, Landshut, Germany).

**Results:**

Patients treated with DAA showed significantly higher inclination (SP: 39.7° ± 7.3° vs. DAA: 44.7° ± 5.3°) and significantly lower cup anteversion values (SP: 31.2° ± 7.9° vs. DAA: 27. 5° ± 5.3°, *p* < 0.001) than patients undergoing THA via SP postoperatively. The horizontal femoral offset was neither preoperatively nor postoperatively higher in DAA than in SP cohort (preoperative: *p* = 0.71, postoperative: *p* = 0.25) (preoperative: SP:37.2 mm ± 7.3 vs. DAA 38.2 mm ± 7.5; postoperative: SP: 38.0 mm ± 7.2 vs. DAA: 40.5 mm ± 7.0). At both times, the acetabular offset was significantly higher in DAA cohort than in SP cohort (preoperative: SP: 32.9 mm ± 5.9 vs. DAA: 36.8 mm ± 4.9; postoperative: SP: 28.9 mm ± 4.2 vs. DAA: 33.4 mm ± 3.8) (preoperative: 0.001; postoperative: *p* < 0.001). The vertical height was preoperatively and postoperatively not significantly higher in SP cohort than in DAA cohort (preoperative: SP: 16.1 mm ± 4.1 vs. DAA: 15.5 mm ± 4.9; postoperative: SP: 16.6 mm ± 4.6 vs. DAA: 16.1 mm ± 4.6) (preoperative: *p* = 0.77; postoperative: *p* = 0.58). The preoperatively existing leg length discrepancy of the affected leg could be compensated via surgery without showing significant differences between the two cohorts (preoperative: SP: −3.2 mm ± 5.4 vs. DAA: 1.9 mm ± 4.9; postoperative: SP: 1.5 mm ± 5.4 vs. DAA: 4.8 mm ± 5.6) (preoperative: *p* = 0.34; postoperative: *p* = 0.09).

**Conclusion:**

The current study demonstrates suitable cup positioning and stem alignment in the coronal plane using minimal-invasive approaches DAA and Superpath®.

## Abstract (de)

**Hintergrund:**

Das Ziel dieser Studie war es die radiologischen Parameter von Patienten, die minimal-invasiv über einen direkten anterioren Zugang („direct anterior approach“ [DAA]) oder SuperPath® (Microport Orthopedics, Arlington, Virginia, USA) operiert wurden, zu vergleichen.

**Material und Methoden:**

Zwischen Januar 2018 und Februar 2019 wurden prä- und postoperative radiologische Parameter von 80 Patienten, die sich einer Hüftendoprothesenimplantation über die beiden minimal-invasive Zugänge DAA (*n* = 40) und SP (*n* = 40) unterzogen, erhoben. Die radiologische Auswertung wurde mit der digitalen Software mediCad® (HECTEC™ GmbH, Landshut, Germany) vorgenommen. Die erhobenen radiologischen Parameter waren die Pfannenanteversion, die Pfanneninklination, das Drehzentrum, die vertikale Höhe, das vertikale femorale Offset, das horizontale femorale Offset, das azetabuläre Offset und die Beinlängendifferenz.

**Ergebnisse:**

Patienten, die über einen DAA operiert wurden, zeigten signifikant höhere Pfanneninklinationswinkel (SP: 39,7° ± 7,3° vs. DAA: 44,7° ± 5,3°; *p* = 0,001) als Patienten, die über einen SP-Zugang operiert wurden. Die Pfannenanteversion war signifikant niedriger in der DAA-Gruppe als in der SP-Gruppe (SP: 31,2° ± 7,9° vs. DAA: 27,5° ± 5,3°; *p* = 0,001). Das horizontale femorale Offset war weder prä- noch postoperativ signifikant höher in der DAA-Gruppe als in der SP-Gruppe (präoperativ: SP: 37,2 mm ± 7,3 mm vs. DAA 38,2 mm ± 7,5 mm; postoperativ: SP: 38,0 mm ± 7,2 mm vs. DAA: 40,5 mm ± 7,0 mm, präoperativ: *p* = 0,71, postoperativ: *p* = 0,25). Prä- und postoperativ war das azetabuläre Offset in der DAA-Gruppe signifikant höher als in der SP-Gruppe (präoperativ: SP: 32,9 mm ± 5,9 mm vs. DAA: 36,8 mm ± 4,9 mm; postoperativ: SP: 28,9 mm ± 4,2 vs. DAA: 33,4 mm ± 3,8 mm; präoperativ: *p* = 0,001; postoperativ: *p* < 0,001). Die vertikale Höhe war prä- und postoperativ nicht signifikant höher in der SP-Gruppe als in der DAA-Gruppe (präoperativ: SP: 16,1 mm ± 4,1 mm vs. DAA: 15,5 mm ± 4,9 mm; postoperativ: SP: 16,6 mm ± 4,6 mm *vs* DAA: 16,1 mm ± 4,6 mm, präOP: *p* = 0,77; postOP: *p* = 0,58). Die präoperativ bestehende Beinlängendifferenz konnte durch die Operation in beiden Gruppen ausgeglichen werden, ohne jedoch signifikante Unterschiede zwischen den beiden Gruppen zu zeigen (präoperativ: SP: −3,2 mm ± 5,4 mm vs. DAA: −1,9 mm ± 4,9 mm; postoperativ: SP: 1,5 mm ± 5,4 mm vs. DAA: 4,8 mm ± 5,6 mm; präoperativ: *p* = 0.34; postoperativ: *p* = 0.09).

**Schlussfolgerung:**

Die aktuelle Studie zeigt eindeutig, dass über die beiden untersuchten minimal-invasiven Zugänge eine akkurate Implantatpositionierung möglich ist.

## Introduction

Total hip arthroplasty (THA) is one of the most common procedures in orthopedic surgery [1] and combines top level patient satisfaction with low complication rates [2]; however, traditional surgical techniques are associated with undesirable side effects including extensive skin incision, tissue damage, increased perioperative blood loss and delayed postoperative rehabilitation [3]. For those reasons, minimally invasive approaches, such as the direct anterior approach (DAA), have been introduced into THA [4]. First short-term results of DAA demonstrated promising results which were attributed to intermuscular approach [5]. Yet, there are several important problems with minimal-invasive approaches which have to be concerned, such as prolonged learning curves, posterior dislocation and component malposition due to limited overview and extensive posterior soft tissue release [6–10].

In 2011, an innovative minimal-invasive surgical technique, SuperPath® (supercapsular percutaneously assisted total hip), was introduced into THA [11]. The method unites the percutaneously assisted total hip (PATH, Wright Medical Technology, Memphis, TN, USA) and supercapsular (SuperCap, Wright Medical Technology) methods. First studies investigating SuperPath® approach showed encouraging results with shorter length of hospitalization stay and accurate implant positioning [7, 12–15]. The complication rates following joint replacement by the SuperPath® technique were described as very low [11, 16, 17].

The purpose of the present study was to compare the postoperative radiographic results of SuperPath® and DAA as minimal-invasive approaches in THA.

## Material and methods

Between January and December 2018, 132 patients received THA due to primary arthritis using a minimal-invasive approach in a maximum care endoprosthesis center. Of the patients 80 could be contacted by telephone and gave the written consent to participate. The study excluded patients with femoral neck fractures, femoral head necrosis and posttraumatic arthritis as indications for hip joint replacement. Further exclusion criteria were the implantation of short stem femoral implants and cemented femoral stem implants. The medical history was collected by file research and the radiographic evaluation was made by two experienced surgeons. The observers were blinded and were not involved in the surgery.

### Surgical techniques and evaluation of intraoperative data

All surgeries were performed by two experienced hip surgeons. MH used the DAA while DB performed THA via SP. A digitalized planning tool (mediCad®, HECTEC™ GmbH, Landshut, Germany) was used for preoperative planning. All patients underwent general anesthesia and received a single-shot antibiotic prophylaxis (cefazolin 2 g) before incision.

The patients in the DAA group were positioned supine on the operating table. The hip was positioned at the table break in order to allow extension during the procedure. The proximal starting point of skin incision was determined ~2 fingerbreadths lateral and ~1 fingerbreadth distal from anterior superior iliac spine (ASIS). A skin incision of suitable length was made along the straight line connecting proximal starting point and fibula head. The interval between the tensor fascia latae and the sartorius was opened and the circumflex vessels were coagulated. The anterior joint capsule could be visualized and incised longitudinally. A slight release of the proximal lateral hip capsule at the acetabular rim allowed a visualization of the joint space. A retractor can be positioned adjacent to the anterior inferior iliac spine (AIIS) to make medial joint space visible. To protect the trochanteric region another retractor was positioned next to the lateral femoral neck. From the exposed neck, a V-shaped piece of bone was cut out and the femoral head was removed. After head removal, the acetabulum could be directly inspected at the base of exposure. Anterior osteophytes and parts of the labrum were resected. The acetabulum was milled gradually in ascending order until all remnants of the cartilage are removed and microbleeding of the bone was visible. A press fit uncemented acetabular cup was then placed. If necessary, screws were inserted into acetabular cup. After palpatory and visual examination of the cup position, a polyethylene liner was impacted. To get access to the proximal femur, the leg was positioned with extension, external rotation and adduction in the ipsilateral hip joint. A slight knee flexion facilitates the external rotation. The remaining posterior joint capsule tissue was removed so that superior aspect of the femoral neck and piriformis fossa could be inspected. The release of remaining joint capsule soft tissue adhering to the femoral neck was described as crucial by some authors for obtaining appropriate access to proximal femur [18]. The femoral shaft should be external rotated for at least 90°. The proximal femur was closely positioned lateral to the acetabular component. The femoral neck was elevated with a support hook (femoral elevator) so that the access to femoral canal could be obtained using a box cutting osteotome.

**Fig. 1:**
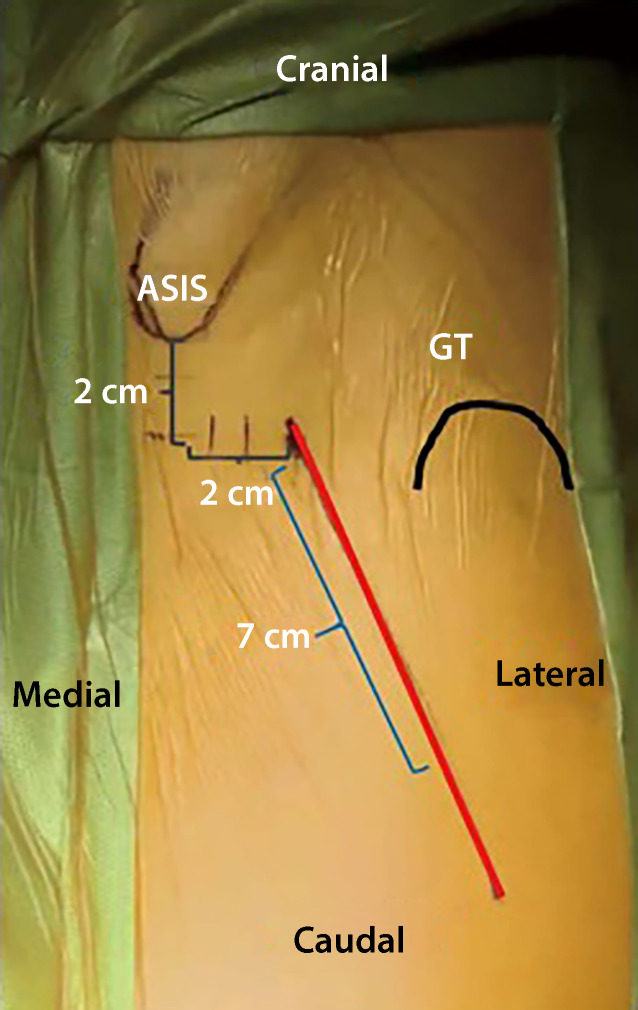
Photograph from a surgical site showing anatomical landmarks and incision line in DAA (direct anterior approach). (From Connolly and Kamath [21]). *ASIS* anterior superior iliac spine, *GT* greater trochanter

The preparation of the femoral canal was done with appropriate version and lateralization gradually in ascending order until press fit was reached. After trial reduction and careful fluoroscopy to evaluate the canal fit and offset of the femoral component, the femoral trial components was removed and the situs was irrigated carefully. If trial reduction showed no tendency of dislocation and an accurate range of motion, femoral component was implanted. After implantation of femoral component into femoral canal according to recommendation of manufacturer, the cone was cleaned from tissue material by irrigation and following drying with compresses. The final ceramic head was placed and seated. The acetabular component with its polyethylene inlay was irrigated. After reduction, position of components was assessed with final fluoroscopy and the stability of the hip was confirmed manually ([19, 20]; Fig. 1).

**Fig. 2:**
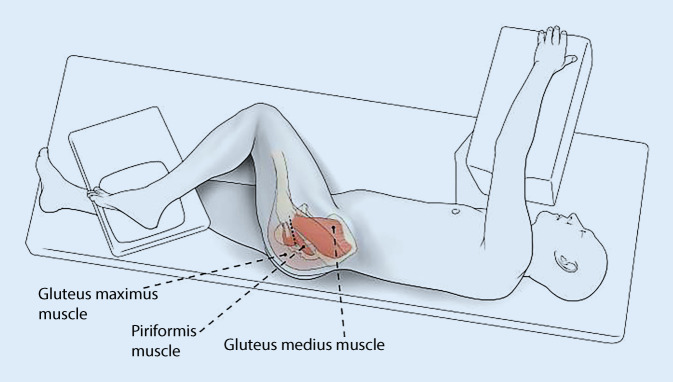
Diagram showing Patient positioning and incision line of SuperPATH® approach. (From Lovell [20], Quitmann [22])

In the SuperPath® group, the patients were positioned in the standard lateral decubitus position with the involved leg in the “home position”, i.e., 45°–60° of flexion, 20°–30° of internal rotation, and slight adduction by elevating the foot on a padded Mayo stand according to Chow (2011) [11]. A skin incision of suitable length was done from the tip of the trochanter in line with femur axis in proximal direction. After transection of subcutis, fibers of the gluteus maximus were split in line with the skin incision. The bursa tissue has to be incised at the posterior rim of gluteus medius. The gluteus medius was retracted in anterior direction to identify the piriformis tendon. The gluteus minimus was dissected and moved anterior under use of Cobb elevator. After elevating the ipsilateral knee, sharp retractors were placed under piriformis tendon posterior and gluteus medius to visualize the joint capsule. After incision of joint capsule in line with the skin incision using an electrocautery, the labrum of the acetabulum was partially removed. At this point, hemostasis at the basis of the capsule was crucial [22]. The sharp elevators were removed and blunt Hohmann retractors were inserted around the neck, first posterior then anterior. After identifying the saddle of femoral neck as point of entry, the femoral preparation started with opening of the femoral canal using a sharp start reamer. At this point, it was important to confirm intramedullary reaming with a cortical feeler gauge. A channel was reamed at the upper margin of femoral neck from the femoral canal to the center of femoral head. Then, the femoral canal was broached in ascending order under protection of femoral head and neck. With the broach as a saw guide neck osteotomy was determined. After neck resection, a Romanelli retractor was placed inside the capsule just at the anterior and posterior wall to explore the acetabulum. After identifying the transverse ligament, the rest of the acetabular labrum was removed. To release the tension of the sciatic nerve, the leg was placed with flexed knee and extended hip. After palpation of the femur, a 1 cm incision was done 1–2 cm posterior to the femur that allowed the creation of a cannula through the gluteus maximus muscle ending posterior to the femoral neck inside the joint capsule.A bone hook was placed into the shoulder of the femoral broach to retract the femur anterior if necessary. Acetabular preparation and cup impaction were performed via a portal without needing release of the iliotibial tract or remaining external rotators using a sharp Romanelli self-retaining retractor (Innomed, Savannah, GA, USA) and a modified Zelpi selfretaining retractor (Life Instruments, Braintree, MA, USA). After cup impaction and inlay insertion trial reduction was performed under the use of a bone hook and a T-handle to gently manipulate the femur ([23]; Abb. 2).

A bone hook is placed into the shoulder of the femoral broach to retract the femur anterior if necessary. Acetabular preparation and cup impaction are performed via a portal without needing release of the iliotibial tract or remaining external rotators using a sharp Romanelli self-retaining retractor (Innomed, Savannah, GA, USA) and a modified Zelpi self-retaining retractor (Life Instruments, Braintree, MA, USA). After cup impaction and inlay insertion trial reduction is performed under the use of a bone hook and a T-handle to gently manipulate the femur [23].

All patients received press fit acetabular and femoral components. In the DAA group, the acetabular component was a cementless fixed Plasmacup DC® (Aesculap AG, Tuttlingen, Germany) which is a hemispheric cup with a microporous titanium coating (Plasmapore®). The femoral stem types used in the DAA group were Trendhip® (Aesculap AG). The bearing surfaces were ceramic heads on vitamin‑E blended highly cross-linked ultra-high molecular weight polyethylene inlays (UHMWPE-XE, Vitelene®). The head diameter ranged from 28–36 mm.

In Superpath group, all patients received Procotyl L® (Microport Orthopedics Arlington, TN, USA) cups with ultra-high molecular weight polyethylene (UHMWPE) inlays sized 28–36 mm internal diameter. Profemur L® (Microport Orthopedics) with a ceramic head was used in all patients of Superpath group as femoral stem.

## Postoperative treatment and follow-up

All patients got therapeutic dose of low molecular weight heparin as thromboembolism prophylaxis for a minimum of 5 weeks. All patients received physiotherapy and were mobilized 1 day after surgery. The amount of weight bearing was dependent on individual decision of each surgeon and varied from full weight-bearing immediately after surgery to at least 20 kg weight-bearing for 6 weeks.

## Radiographic measurements

We used preoperative and 7‑day postoperative digitalized radiographs with an anteroposterior and lateral view. For the templating and measurements, we used a digital image analysis system (mediCad™, HecTec GmbH, Niederviehbach, Germany). Scaling is carried out via 3‑point scale with a coin of standard size as reference level which positioned on imaging plate.

The hip center of rotation (COR) was specified as the center of a circle drawn around the edge of the femoral head and its center [24–26]. The horizontal femoral offset (HFO) was determined as the perpendicular distance between the COR and the proximal femoral shaft axis (FSA) [17, 18]. The vertical femoral offset (VFO) was defined as the distance between the trans-teardrop line and the medial apex of the lesser trochanter [27]. The vertical height (VH) of the COR was defined as the distance between the preoperative or postoperative COR and the inter-teardrop line [28]. Acetabular offset (AO) was measured as the perpendicular distance between the COR and line at the inner margin of ipsilateral teardrop figure [17, 19, 20]. The limb length differences were then determined by measuring the perpendicular distance (mm) between the teardrop and the apex of the lesser trochanter on either side. The Widmer and McLaren methods were used to evaluate the cup inclination and anteversion [29–31]. The caput-collum-diaphyseal angle (CCD angle) was determined as the angle between the proximal femoral shaft axis and the axis of the femoral neck (preoperative) and the neck of the prosthesis (postoperative).

## Statistical analysis

Summary statistics of the data were expressed as mean ± SD. The Shapiro-Wilk test was used to test for normal distribution. The paired Student’s *t* test was used for comparison of the normal distribution of preoperative and postoperative means and the Wilcoxon signed-rank test for non-normal distribution. The comparisons with *p*-values < 0.05 were considered to be significant. The software SPSS 19 (IBM, Armonk, NY, USA) was used to carry out the statistical computations.

## Results

### Patient demographics

In the present study, postoperative radiographic images of 80 patients were evaluated. The evaluated demographics were homogeneous in both groups (Table 1). In the DAA group, gender ratio was 16:24 (male:female) and 9:31 in the in the SuperPath® group. The mean age at the time of arthroplasty was 68.6 years ± 8.7 (48–82) in the DAA group and 68.8 years ± 8.7 (51–85) in the Superpath® group (*p* = 0.92). The average body mass index (BMI) was measured 26.1 kg/m^2^ ± 2.1 (22–30) and 25.2 kg/m^2^ ± 1.8 (21–30) in the SuperPath® group (*p* = 0.06).

**Table 1:** Radiographic parameters preoperatively and postoperatively

Parameter	SP preop	DAA preop	*p*-value	SP postop	DAA postop	*p*-value
Inclination (°)	–	–	–	39.7 ± 7.3	44.7 ± 5.3	0.001
Anteversion (°)	–	–	–	31.2 ± 7.9	27.5 ± 5.3	0.001
Vertical femoral offset (mm)	–	–	–	44.0 ± 9.2	49.4 ± 7.1	0.001
Horizontal femoral offset (mm)	37.2 ± 7.3	38.2 ± 7.5	0.71	38.0 ± 7.2	40.5 ± 7.0	0.25
Acetabular offset (mm)	32.9 ± 5.9	36.8 ± 4.9	0.001	28.9 ± 4.2	33.4 ± 3.8	<0.001
Vertical height (mm)	16.1 ± 4.1	15.5 ± 4.9	0.77	16.6 ± 4.6	16.1 ± 4.6	0.58
Leg length discrepancy (mm)	−3.2 ± 5.4	−1.9 ± 4.9	0.34	1.5 ± 5.4	4.8 ± 5.6	0.09
CCD (°)	132.9 ± 10.8	131.7 ± 8.1	0.55	133.3 ± 6.7	131.0 ± 19.4	0.65

### Radiographic evaluation

**Fig. 3:**
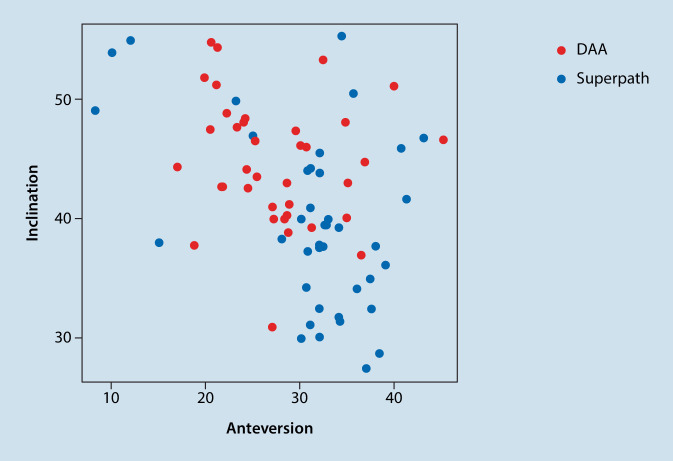
Scatterplot of inclination and anteversion between DAA (direct anterior approach) and SuperPath

**Fig. 4:**
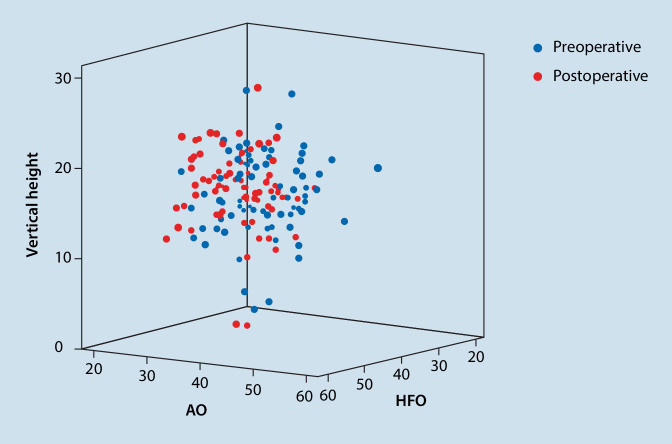
COR Reconstruction pre- and postoperative over with both techniques. (own data) *AO* acetabular offset, *HFO* horizontal vertical offset, *COR* center of rotation reconstruction pre- and postoperative with both techniques, *VH* vertical height

**Fig. 5:**
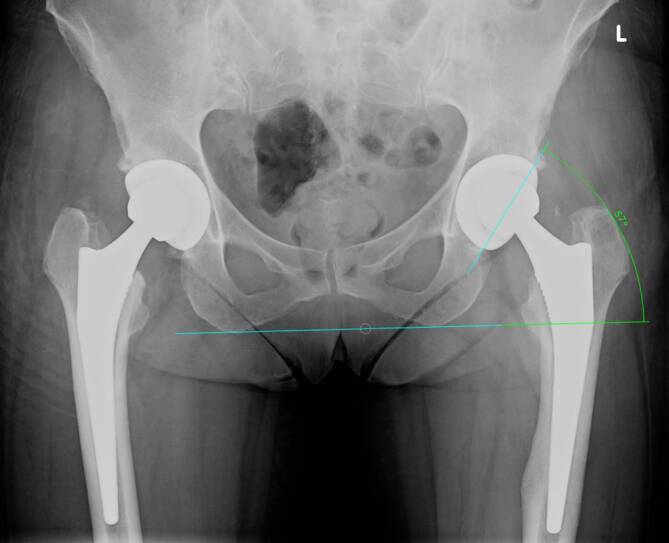
Postoperative radiograph from a patient who underwent THA via DAA on the left side. The digital image analysis reveals a high inclination value

**Fig. 6:**
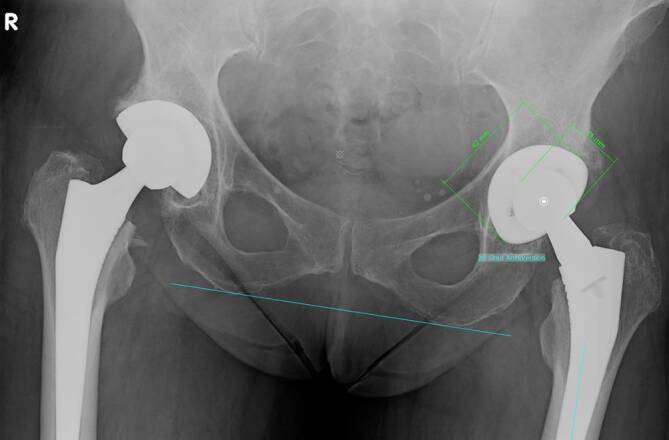
Postoperative radiograph from a patient who underwent THA via SP on the left side. The digital image analysis reveals a high anteversion value

**Fig. 7:**
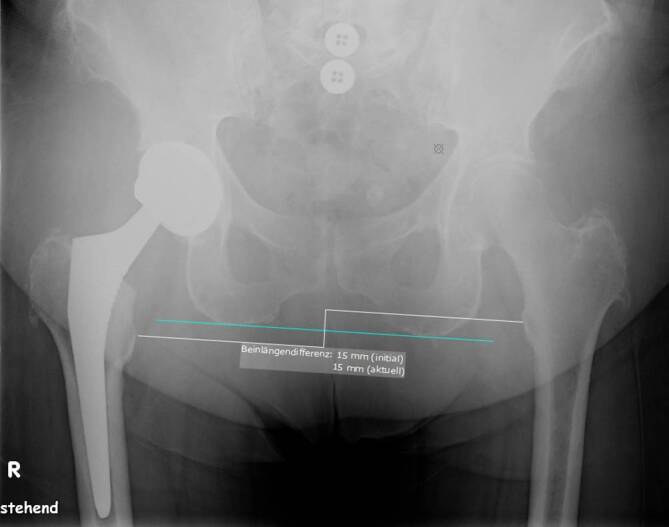
Postoperative radiograph from a patient who underwent THA via DAA. The digital image analysis shows leg length discrepancy of 15 mm

Patients being operated on via DAA showed significantly higher inclination and lower anteversion values than patients from the SP cohort (*p* = 0.001) (see Table 1 and Fig. 1). Patients from DAA cohort had preoperatively and postoperatively non-significantly higher horizontal femoral offset levels (preoperative: *p* = 0.71; post: *p* = 0.25). At both times, patients who were treated via DAA had significantly higher acetabular offset values (preoperative: *p* < 0.001; postoerative: *p* < 0.001). The values for vertical height were at both times non-significantly higher in SP cohort than in DAA cohort (preoperative: *p* = 0.77; postoerative: *p* = 0.58). The leg length reduction of the affected limb converted to increased leg length postoperatively without showing significant differences between the two cohorts (preoperative: *p* = 0.34; postoerative: *p* = 0.09). There was no difference in CCD pre- and postoperatively between the two cohorts (preoerative: *p* = 0.55; postoerative: *p* = 0.65). Fig. 3 shows scatterplot of inclination and anteversion in DAA (direct anterior approach) and SP (SuperPath®). Fig. 4 illustrates COR Reconstruction pre- and postoperative over with both techniques. Figs. 5, 6 and 7 show postoperative radiographs from patients who underwent MIS (minimal-invasive surgery) THA via DAA (direct anterior approach) or SuperPath®.

## Discussion

The application of highly specialized minimally invasive surgical approaches in THA has increased over the last years [32, 33]. Especially, the direct anterior approach (DAA) has gained in popularity [34]. A meta-analysis by Higgins et al. (2015) about the clinical and surgical results of THA via DAA revealed better results regarding postoperative pain, functional outcomes, length of hospitalization, hip stability and postoperative narcotic consumption in comparison with traditional posterior approaches [35, 36]. Similarly, the PRISMA meta-analysis comparing DAA with lateral approach showed superior results of DAA with regard to early postoperative functional rehabilitation, lower levels of perceived pain, and shorter hospitalization time [37]; however, some serious complications such as higher rates of posterior dislocation due to implant malposition and posterior soft tissue release were reported in association with DAA [34–36].

Recently, SuperPath® was introduced as an alternative minimal-invasive approach in THA [11]. The first reports about clinical and radiographic results of SuperPath® were highly promising with accurate implant positioning and low complication rates [12–18].

The purpose of the current study was to reveal the differences in postoperative radiographic outcome of two minimal-invasive surgical approaches in THA (DAA vs. SuperPath®).

Hip dislocation is a frequent complication after THA. It is associated with a multitude of reasons [38, 39]. Most hip dislocations occur in the immediate postoperative period (30–60%) [40–42]. Acetabular cup positioning seems to be crucial for hip joint stability and function after THA. Inclination and anteversion angles are reliable parameters to assess postoperative cup positioning in THA [43]. The “safe zone” first described by Lewinnek et al. (1978), indicates the critical values for inclination (30–50°) and anteversion (5–25°) [44]; however, the “death” of the zone was recently proclaimed by Tezuka et al. (2019) with the hypothesis that patient’s individual functional stability is more important to prevent dislocation than rigid limiting values [45]. Rasuli et al. (2015) reported in an article about the radiographic outcomes of the PATH and SuperPath® approaches that cups were significantly more anteverted in the SuperPath® cohort without increased dislocations rates [46]. In the current study, in SP cohort there were significantly higher anteversion and significantly lower inclination angles in comparison with DAA (see Table 1 and Fig. 1). The impact of these is still not fully clear. We know that higher inclination leads to higher edge loading and wear rates and that a higher anteversion leads to higher dislocation rates to the front and lower anteversion to the back [47]. In our data, the inclination and anteversion differ between the two techniques but is in mean within the safe zones from the literature and there were no cases of dislocation. A possible explanation for the significant differences in the anteversion and inclination values could be the different position of the patients on the operating table. In DAA the patients are place in supine position and in SP in standard lateral decubitus position.

In several studies it was demonstrated that dislocation is associated with superolateral or inferolateral position of COR at distances > 5 mm away in comparison with the native COR [8, 48, 49]. Our data demonstrate that the COR was equally reconstructed with both techniques (Fig. 2). The significant difference in the acetabular offset (AO) is explained with the also existing preoperative significant difference (Table 1). With this in mind it would be of concern if the preoperative difference could not be found postoperatively. All other parameters regarding the COR were not statistically significant.

Leg length discrepancy (LLD) is a common cause for patient dissatisfaction after THA [35] and frequently causes gait disorders, nerve injury, lower back pain and hip instability. The current data situation concerning LLD is not clear. There are reports on LLD up to 10 mm without symptoms while others claimed that even small discrepancies could produce dissatisfaction [9, 35]. One of the first reports about radiographic outcome of SuperPath® in 150 patients by Quitmann (2019) did not reveal LLD above 5 mm in any patient [20]. There are only a few data on LLD in DAA available. Lv et al. (2017) and Lee et al. (2017) reported on LLD in patients treated with DAA not exceeding 5 mm [10, 50]. In the current study, patients treated via DAA had an LLD from 4.8 ± 5.6 mm and patients treated via SuperPath® 1.5 ± 5.4 mm. Accordingly there was no statistical difference between both treatments regarding the LLD (*p* = 0.09). In total, our results are in accordance with the current literature  demonstrating good reconstruction of LLD with DAA and SuperPath®.

Stem alignment is usually placed neutrally and parallel to the femoral shaft axis. In contrast, intraoperative stem malpositioning in the coronal plane may affect offset or leg length restoration and can impair optimum load transfer between the implant and the natural bone [51]. Especially, varus stem alignment can lead to femoral cortical hypertrophy and thigh pain [52]. Low centrum-collum-diaphysis(CCD)-angles, a long neck and a trochanter overhang enhance the risk for intraoperative varus stem positioning [53]. In our study, we used the Trendhip® (Aesculap AG, Tuttlingen, Germany) and the Profemur L® (Microport Orthopedics Arlington, TN, USA) stems. The Trendhip® stem which was used in DAA has a CCD angle of 134°. The Profemur L® stem being used SP has a CCD angle of 135°. Neither preoperatively nor postoperatively could we find significant differences between the two cohorts. The slightly higher CCD angles in SP cohorts might results from the implant design with 1° higher CCD angle of the Profemur® L stem.

One of the major problems of DAA is the limited overview due to small skin incision length. To get an accurate access to the femoral canal, it is often necessary to elevate the femur with previous slight release of posterior joint capsule and muscle tendons from the femoral bone [54]. As a result, the risk of posterior dislocation might be increased, if damage of posterior structures is too large [55, 56]. Especially, the short external rotators have to be preserved to provide dorsal hip stability [57, 58]. For this reason, capsule and posterior muscle tissue-preserving techniques were devised. In these techniques, the posterior joint capsule is cut in a proximal-distal direction at the midpoint of the attachment site on the femur and the attachment site on the posterior acetabular roof [31, 50, 59–62].

We acknowledge that the current study has several limitations. Firstly, this is a retrospective control cohort study. The bias in patient selection, lack of randomization, and difference in operative techniques between surgeons might exist in this study. Secondly, the use of plain radiographs rather than CT scans to measure cup positioning may have led to slight variations based on patient pelvic tilt and rotation.

A major strength of our study is that we report for the first time on a comparison between the recently introduced MIS hip approach (SuperPath®) and the established MIS-DAA approach. Furthermore, surgery was performed by only two experienced hip surgeons from a level one endoprosthetic center. Both surgeons are familiar with minimal-invasive approaches in THA for years. The impact of potential differences due to age, gender and BMI has been considered. Only patients with osteoarthritis were included as other diagnoses such as rheumatoid arthritis and femoral neck fractures were excluded.

## Conclusion

Regardless of the used minimal-invasive technique (DAA/SuperPath®) it is possible to achieve suitable cup positioning and stem alignment in the coronal plane. The restoration of center of rotation was feasible with both minimal-invasive approaches. Neither DAA nor SP provoked leg length discrepancy. The impact of this observation on the durability of the prostheses needs to be investigated in the future by long term follow-up studies.

## List of Abbreviations

AIIS: Anterior inferior iliac spine
AO: Acetabular offset
ASIS: Anterior superior iliac spine
BMI: Body mass index
CCD: centrum-collum-diaphyseal
COR: Center of rotation
CT: Computer tomography
DAA: direct anterior approach
HFO: Horizontal femoral offset
LLD: Leg length discrepancy
MIS: Minimal invasive surgery
ROM: Range of motion
SD: Standard deviation
SP: SuperPATH®
THA: Total hip arthroplasty
VFO: Vertical femoral offset
VH: Vertical height
